# Response of Soil Microbial Communities to Karst Desertification in Soil and Water Conservation Agroforestry Systems

**DOI:** 10.3390/microorganisms14030556

**Published:** 2026-02-28

**Authors:** Wanmei Hu, Kangning Xiong, Anjun Lan, Min Zhang, Liheng You, Jifeng Zhang, Zhenquan Zhong

**Affiliations:** 1 School of Karst Science, State Key Laboratory Cultivation Base for Guizhou Karst Mountain Ecology Environment, Guizhou Normal University, Guiyang 550025, China; 21030170042@gznu.edu.cn (W.H.);; 2 School of Geography and Environmental Science, Guizhou Normal University, Guiyang 550025, China

**Keywords:** agroforestry, soil and water conservation, soil microorganisms, KD, soil physicochemical properties, karst, bacteria, fungi

## Abstract

Karst desertification (KD) severely constrains regional ecological security and sustainable development. As an important ecological restoration measure, soil and water conservation agroforestry (SWCAF) systems have unclear mechanisms for soil microbial responses. This study investigated the effects of potential–light (PL), light–moderate (LM), and moderate–high (MH) KD on soil physicochemical properties and microbial communities in Karst SWCAF (KSWCAF) systems. It explored the drivers of microbial community changes. The results showed that (1) Soil physicochemical properties exhibited nonlinear changes along the KD gradient. Key soil-fertility indicators including silt, clay, total porosity (TP), total phosphorus (Total_P), total nitrogen (Total_N), soil organic carbon (SOC), and carbon nitrogen ratio (C_N) showed significant unimodal patterns, peaking at the LM stage with optimal overall soil quality; (2) The dominant bacterial phyla were *Pseudomonadota*, *Acidobacteriota*, *Actinomycetota*, and *Planctomycetota*, while the dominant fungal phyla were *Ascomycota*, *Basidiomycota*, and *Mortierellomycota*. The overall abundance of these dominant phyla increased with intensifying KD, except that the relative abundance of *Pseudomonadota* was lowest in the QZ study area, while *Acidobacteriota* was highest in the QZ area. The dominant fungal phylum *Ascomycota* increased with KD intensification; (3) KD significantly influenced microbial community structure and beta diversity. Fungi showed stronger responses to the KD gradient than bacteria. Bacterial alpha diversity was significantly higher in the LM stage compared to the PL and MH stages (*p* < 0.05), while fungal alpha diversity was significantly lowest in the MH stage (*p* < 0.05); (4) Bacterial networks exhibited highest complexity but reduced stability at the LM stage, whereas fungal networks enhanced stability at the MH stage by increasing modularization and positive correlation proportions; (5) RDA revealed that soil physicochemical factors explained 66.89% and 98.82% of bacterial and fungal community variation, respectively, with pH, moisture, and C_N as key drivers. Overall, KD regulates microbial community structure and functional allocation by reshaping the soil environmental gradient, with the LM stage potentially representing a “transitional optimization window” for KSWCAF ecosystem structure and function. This study provides a theoretical basis for microbial regulation strategies in KD control and soil and water conservation (SWC) processes.

## 1. Introduction

Karst desertification (KD) is among the most severe forms of land degradation in karst regions worldwide. It disrupts soil structure, reduces organic carbon content, intensifies drought stress, and drives a self-reinforcing cycle of vegetation degradation, soil erosion, and ecosystem collapse. This process results in substantial loss of land resources and exacerbates regional poverty, posing a major constraint on sustainable development in karst areas [1,2]. As central regulators of biogeochemical cycling and ecosystem functioning, soil microorganisms directly mediate organic matter decomposition, nutrient transformation (e.g., carbon, nitrogen, and phosphorus cycling), and soil structural stability [3,4,5]. Increasing evidence indicates that KD not only alters soil physicochemical properties but also profoundly reshapes soil microbial community structure and ecological functions, thereby accelerating land degradation processes [6]. Elucidating microbial responses to KD and clarifying their regulatory roles in degraded karst ecosystems are, therefore, critical for advancing ecological restoration and land management strategies.

Soil constitutes the most biodiverse terrestrial habitat on Earth [7], harboring immense microbial diversity [8,9]. It is estimated to contain more than 10^30^ microbial cells, forming one of the most complex biological networks on the planet [10]. Soil microbial communities—primarily composed of bacteria, fungi, and archaea [11]—serve as key indicators of ecosystem recovery. Through processes such as organic matter decomposition, nutrient mineralization, nitrogen fixation, and phosphorus solubilization [12], they maintain soil fertility and underpin essential biogeochemical cycles. In the context of global climate change and intensified anthropogenic disturbances, the ecological significance of soil microbial diversity has received increasing attention. Studies have been conducted across diverse ecosystems, including global drylands [13], saline–alkaline soils [14,15], the Qinghai–Tibet Plateau [16,17,18], the Mediterranean Basin [19], and karst landscapes [20,21]. These investigations span major ecosystem types such as forests [22], grasslands [23,24,25], wetlands [26,27], croplands [28,29,30], and agroforestry systems (AF) [31,32,33]. Methodologically, the field has progressed from traditional microscopy-based approaches to advanced molecular techniques, including quantitative PCR [34,35], amplicon sequencing [34], and metagenomic analyses [16,26], which have revealed pronounced spatial heterogeneity in microbial community composition across soil types and land-use regimes [36].

Agroforestry has emerged as a promising ecological restoration strategy for mitigating land degradation by improving microenvironmental conditions and enhancing soil properties [37,38], thereby integrating ecological and economic benefits [39,40]. Since early quantitative assessments demonstrated that tree rows increase soil bacterial and fungal abundance in temperate AF [41], research in this field has expanded rapidly. A broad consensus now recognizes that soil microbial communities in AF are jointly regulated by multiple interacting factors, including tree species identity [42], spatial configuration [42], soil moisture [31], temperature [43], porosity [44], organic matter content, and pH [45]. However, studies specifically examining soil microbial communities and their environmental drivers in karst soil and water conservation agroforestry systems (KSWCAF) remain limited.

Karst landforms are widely distributed around the world and are recognized as typical fragile ecosystems [46,47]. For instance, in karst regions of Africa, the Middle East, and the Mediterranean, drought stress combined with irrational human activities has led to intensifying land degradation and a continuous loss of ecosystem services [48,49]. Similarly, in the karst areas of Barbados and Haiti, overexploitation of land and unsuitable agricultural practices have resulted in vegetation degradation, declining soil quality, and ecosystem imbalance, ultimately leading to the deterioration of ecosystem functions [49]. The karst area in southern China, centered on the Guizhou Plateau, constitutes the largest and most contiguous karst region among the world’s three major karst distribution zones, covering an area exceeding 55 × 10^4^ km^2^ [50]. In this region, KD is the most critical ecological and environmental issue, severely constraining both ecological integrity and socioeconomic development [1]. Controlling KD has become a primary challenge and a key focus of ecological restoration efforts in Guizhou Province [51].

To address this issue, KSWCAF systems have been implemented as key projects to restore ecosystems degraded by KD. These measures—such as closing hillsides to facilitate forest regeneration, establishing silvopasture systems, alley cropping, converting sloping farmland to terraces, and developing basic farmland—play a vital role in advancing ecological restoration and supporting regional economic development. KSWCAF represents an integrated land-use system specifically adapted to the challenging conditions of karst terrain, including steep slopes, shallow and patchy soils, low fertility, and water scarcity. It typically arranges conservation measures along topographical gradients from hilltops to valleys, combining afforestation, silvopasture, hedgerows, and improved farmland.

Although extensive research has been conducted on karst ecosystems [52,53,54,55], studies focusing on soil microorganisms in karst regions remain relatively scarce. Existing research has primarily addressed ecosystems such as karst forests [21,56], grasslands [57], croplands, and moss crusts [58,59,60,61]. Some attention has also been given to specialized karst microenvironments, including karst Tiankengs [62] and positive/negative topographies [63], and their effects on microbial communities. However, research addressing microbial-based approaches to KD control [64] and the specific impacts of KD on soil microorganisms remains limited. Moreover, studies on karst agroforestry ecosystems have mainly reported changes in soil physicochemical properties, such as moisture and nitrogen content [65,66]. In contrast, the characteristics of soil microbial communities and their relationships with soil properties within KSWCAF systems have not been systematically investigated.

To address these knowledge gaps, this study selected three typical environments in the karst region of southern China, representing potential–light (PL), light–moderate (LM), and moderate–high (MH) grades of karst desertification, as research sites. Focusing on typical KSWCAF measures—including closing hills for forest regeneration, silvopasture, alley cropping systems, and basic farmland construction—we examined the effects of different KD grades on soil physicochemical properties, microbial community composition, diversity, and co-occurrence network characteristics. Specifically, this study aimed to (1) quantify the effects of KD on soil physicochemical properties in KSWCAF systems; (2) evaluate the impacts of KD on soil microbial communities; (3) analyze the co-occurrence network characteristics of soil microorganisms under different KD grades; and (4) identify the key driving factors underlying changes in soil microbial community structure across KD gradients. The findings are expected to deepen understanding of KD’s impacts on soil microorganisms in AF and to provide a scientific basis for implementing targeted SWC measures (SWC) and guiding ecological restoration efforts in karst regions.

## 2. Research Methods

### 2.1. Study Area Overview

**Figure 1:**
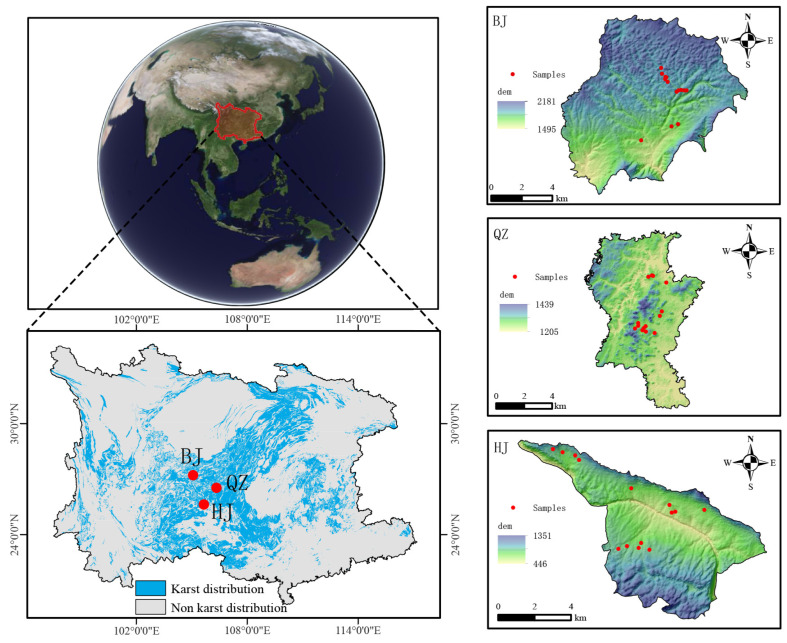
Map of study areas and sampling points.

The study areas are located in Bijie Salaxi (BJ), Qingzhen Hongfeng Lake (QZ), and Guanling-Zhenfeng Huajiang (HJ) in northern, central, and southern Guizhou Province, respectively, within the core karst region of South China (Figure 1). BJ is a typical representative of potential–light karst desertification (PLKD) in southern China (central point coordinates: 27°15′8.5″ N, 105°5′32″ E) [67], with an altitude ranging from 1830 m to 2000 m. This region has four distinct seasons with concurrent rainfall and heat, receiving approximately 1000 mm of annual precipitation, 70% of which falls in summer [65]. QZ is a typical representative of light–moderate karst desertification (LMKD) in southern China (106°18′14″–106°23′24″ E) [67], with an average altitude of approximately 1300 m. The region has an annual mean temperature of about 15 °C and an annual mean precipitation of approximately 1200 mm. SWC measures in QZ primarily include hill closure for forest regeneration, silvopasture, alley cropping, and high-standard basic farmland construction. The HJ study area is dominated by MHKD, with elevations between 450 m and 1450 m, a mean annual temperature of 18 °C, and annual precipitation exceeding 1200 mm [64]. SWC measures in BJ and HJ mainly include hill closure for forest regeneration, silvopasture, alley cropping, and basic farmland formed through slope-to-terrace conversion. The overall thickness of the A-horizon soil in the study area is relatively small, and its spatial distribution is discontinuous. It often appears as patchy patterns interspersed between bare rocks and stone outcrops. Because the parent rock is carbonate rock, the soil gravel content is relatively high, and structural stability is highly dependent on vegetation coverage. Under natural vegetation conditions, the organic-matter content in the surface layer is acceptable. However, due to the limited total soil volume, nutrient and water reserves are low, and the system’s buffering capacity is weak. The A-horizon is extremely sensitive to external disturbances (such as farming, logging, and strong rainfall erosion). Once it is damaged, soil erosion intensifies, and recovery takes a long time. It is a key sensitive layer in the process of ecological degradation and desertification in karst areas.

### 2.2. Soil Sample Collection

In August 2024, four representative plots (10 m × 10 m) were established in each study area, including hill closure for forest regeneration, silvopasture, alley cropping, and basic farmland, for a total of 12 plots. Within each plot, three 5 m × 5 m subplots were set up at both ends and the midpoint of the diagonal for soil sampling (i.e., at least three replicates per agroforestry plot). All subplots in BJ were located in PLKD environments; those in QZ were in LMKD environments; and those in HJ were in MHKD environments. The classification of KD grades was performed following the standard published by Xiong et al. [68] (Table 1). Considering that different land use types significantly affect soil multifunctionality only in the 0–10 cm layer [69], and the shallow soil characteristics of KD, we mainly collected soil at A-horizon for the analysis of soil physicochemical properties and microorganisms. First, the undecomposed litter on the surface layer of each plot was removed. Soil from the 0–10 cm layer was collected using a five-point sampling method, thoroughly mixed, and roots and stones were removed with tweezers. The mixed soil was then quartered diagonally, and two opposite portions were placed into a No. 6 Ziplock bag to obtain one soil sample. Each plot required three replicate mixed samples obtained via five-point sampling at the diagonal endpoints and the midpoint. One additional sampling point was randomly established in each natural forest regeneration and silvopasture plot. Forty-two soil samples for physicochemical property analysis were collected, air-dried naturally in the laboratory, and used for pH, soil texture, SOC, Total_N, Total_K, and Total_P determination. Secondly, within 1 m of each chemical soil sampling point, undisturbed soil cores from the 0–10 cm layer were collected using cutting rings and transported to the laboratory for the determination of soil physical properties and moisture content. Finally, within a range of less than 1 m around each chemical soil sampling point, the undecomposed litter on the surface layer was removed, and soil was excavated from each plot using a sterilized hoe. The soil was thoroughly mixed while wearing sterile gloves, and impurities were removed. The mixed soil was then divided into four portions using the diagonal method, and two portions from opposite corners, totaling 5–10 g, were collected and placed into sterile 50 mL centrifuge tubes. A total of 42 soil-microbial samples were obtained. The samples were stored in an icebox with ice packs, transported to the laboratory, and stored in a −80 °C freezer. Subsequently, they were shipped via dry ice to Guangzhou Genedenovo Biotechnology Co., Ltd. (No. 21 Lixiang 2nd Street, Huangpu District, Guangzhou City, Guangdong Province, China) for soil-microbial analysis.

### 2.3. Soil Sample Experimental Processing Methods

#### 2.3.1. Soil Physicochemical Property Analysis

Soil moisture content (SMC) was determined by oven drying (dried at 105 °C to constant weight). Bulk density (BD) and Total_Porosity (TP) were measured using the cutting ring method [70]. pH was determined by potentiometry. Soil particle size was analyzed using a Bettersize2000 laser particle-size analyzer [71]. The instrument was manufactured by Dandong Better Instruments Co., Ltd., located at No. 9 Ganquan Road, Jinquan Industrial Zone, Lingang Industrial Park, Dandong City, Liaoning Province, China. TP was calculated using the formula: *STP_i_* = 93.947 − 32.995 × *b*, where *b* is bulk density, and *STP_i_* is total porosity [72]. Total_C and Total_N were determined using an elemental analyzer. Total_P was determined by NaOH alkali fusion followed by molybdenum–antimony anti-spectrophotometry. Total_P was determined by alkali fusion with NaOH followed by flame photometry.

#### 2.3.2. Soil DNA Extraction, Bacterial 16S and Fungal ITS Gene Amplification, and Illumina MiSeq Sequencing

Soil fungi and bacteria were determined using high-throughput sequencing of the ITS and 16S regions, respectively [73]. For DNA extraction from soil, 0.25–0.5 g of the soil sample was processed using the HiPure Soil DNA Kits (Magen, Guangzhou, China; Catalog No.: D3142), and DNA purity was detected using a NanoDrop microspectrophotometer (Thermo Fisher Scientific, Waltham, MA, USA). Subsequently, the V3–V4 hypervariable region of the 16S rRNA gene was amplified for soil bacterial DNA using primers 341F (CCTACGGGNGGCWGCAG) and 806R (GGACTACHVGGGTATCTAAT). ITS amplification for soil fungal DNA was performed using primers ITS1_F_KYO2 (TAGAGGAAGTAAAAGTCGTAA) and ITS86R (TTCAAAGATTCGATGATTCAC) targeting the ITS1 region. Third, PCR products were purified using AMPure XP Beads after amplification and quantified with Qubit 3.0 (Thermo Fisher Scientific, Waltham, MA, USA.). Fourth, sequencing libraries were constructed using the Illumina DNA Prep Kit (Illumina, San Diego, CA, USA; Catalog No.: D3142). Fifth, pooling and paired-end sequencing were performed on the Novaseq 6000 platform using PE250 mode (Illumina, San Diego, CA, USA). The specific amplification system and program were as follows: Amplification system included: 10 µL of 5 × Q5@Reaction Buffer, 10 µL of 5 × Q5@ High GC Enhancer, 1.5 µL of 2.5 mM dNTPs, 1.5 µL of Primer F (10 µM), 1.5 µL of Primer R (10 µM), 0.2 µL of Q5@ High-Fidelity DNA Polymerase, X µL (50 ng) of Template, and H_2_O up to 50 µL. Amplification program: 95 °C for 5 min; 30 cycles of 95 °C for 1 min, 60 °C for 1 min, 72 °C for 1 min; and a final extension at 72 °C for 7 min. Strict quality control was conducted after obtaining raw sequencing data, and soil bacterial and fungal OTU sequences were clustered at 100% similarity. The results were processed using DADA2 software (version 1.14.1) to generate ASVs.

### 2.4. Data Processing

Before data analysis, the ASV annotation table was cleaned. After rarefaction based on the minimum sample size, ASVs with total reads less than 42 (n = 42 for all subsamples used in the analysis) were filtered out, ultimately obtaining the dataset for subsequent analysis. One-way ANOVA and multiple comparisons (Tukey-HSD test) in SPSS 21.0, along with the “OmicShare” tool [74], were used to analyze the effects of KD on soil properties, bacterial phylum relative abundance, and bacterial community richness and diversity indices. Stoichiometric characteristic calculations followed [75]. SOC, Total_N, Total_P, and Total_K contents were expressed as mass concentrations, while C_N, C_P, and N_P were expressed as mass ratios [75]. PCoA analysis and a Permutational MANOVA test based on the Bray–Curtis distance were used to compare β-diversity of soil bacteria under different grades of KD in KSWCAF. The R language package was used to visualize the co-occurrence network characteristics of soil microorganisms across different KD grades. Bacterial and fungal functions were predicted using FAPROTAX and FUNGuild tools, respectively. The Variance Inflation Factor (VIF) was used to assess multicollinearity among environmental factors, and those with VIF ≤ 5 were retained (Figure S1) for further exploration of their interactions with microorganisms. Mantel tests were used to analyze the responses of microbial OTUs and the Chao and Shannon indices to soil physicochemical properties. Redundancy analysis (RDA) was used to analyze the correlation between selected environmental factors and soil microbial communities. Spearman correlation heatmaps were used to examine the relationships between environmental factors and soil microbial phylotypes.

## 3. Results

### 3.1. Soil Physicochemical Properties in SWCAF Under Different Grades of KD

Significant differences in soil physicochemical properties were observed among KD grades in SWCAF, with some properties showing distinct trends with increasing KD severity (Table 2 and Table 3).

Soil Physical Properties: KD significantly affected soil physical properties in SWCAF (Table 2). SMC, capillary water holding capacity (CWHC), field capacity (FC), and capillary porosity (CP) ranged from 10.75 to 14.46%, 16.85–19.73%, 15.85–18.23%, and 18.58–21.61%, respectively, all showing an upward trend with worsening KD. Among these, SMC and CP in HJ were significantly higher than in BJ and QZ, while CWHC and FC in QZ and HJ were markedly higher than in BJ (*p* < 0.05). Bulk density (BD), non-capillary porosity (NCP), and Total_Porosity (TP) ranged from 1.09 to 1.11 g/cm^3^, 35.90–38.71%, and 57.28–57.83%, respectively, with no significant differences among different grades of KD (*p* > 0.05). Soil texture also changed with increasing KD severity. Clay and silt contents were highest in QZ and significantly higher than in BJ, but differences were insignificant compared to HJ. Sand content in QZ was considerably lower than in BJ and HJ, with BJ the highest (33.67%), followed by HJ (30.62%), indicating that soil erosion was weakest in the LM desertification environment (QZ), where large amounts of clay and silt were retained. In contrast, PL (BJ) and MH (HJ) desertification environments experienced severe soil erosion, resulting in higher sand content.

Soil Stoichiometric Characteristics (Table 3): Soil pH analysis showed that HJ had a pH of 7.22, significantly higher than QZ (6.70) and BJ (6.46), indicating that higher KD severity leads to a gradual shift from acidic to alkaline soil. Total phosphorus (Total_P) and total nitrogen (Total_N) ranged from 0.74 to 0.92 g/kg and 2.43–2.77 g/kg, respectively, showing a trend of first increasing, then decreasing, with increasing KD grade. However, insignificant differences in Total_P and Total_N were observed across KD grades in KSWCAF, suggesting that Total_P and Total_N levels are primarily influenced by soil parent material and pedogenesis, with no clear relationship to KD grade. Total_K content ranged from 6.06 to 14.56 g/kg and increased with higher KD grade; Total_K in BJ was significantly lower than in QZ and HJ (*p* < 0.05). SOC and C_N ranged from 23.71 to 33.85 g/kg and 9.65–11.99 g/kg, respectively, both showing a trend of first increasing then decreasing with worsening KD, with SOC and C_N in QZ significantly greater than in BJ and HJ. C_P and N_P ranged from 33.69 to 41.58 and 3.13–3.74, showing slight fluctuations with worsening KD but no significant differences. According to the classification standards of the Second National Soil Survey, SOC and Total_K levels across all three KD grades were relatively low, corresponding to the third and fourth levels of soil nutrient status, indicating that potassium fertilizer and organic matter supplementation should be prioritized in karst AF.

### 3.2. Effects of KD on Soil Microbial Communities in KSWCAF

#### 3.2.1. Soil Bacterial and Fungal Community Composition and Relative Abundance

**Figure 2:**
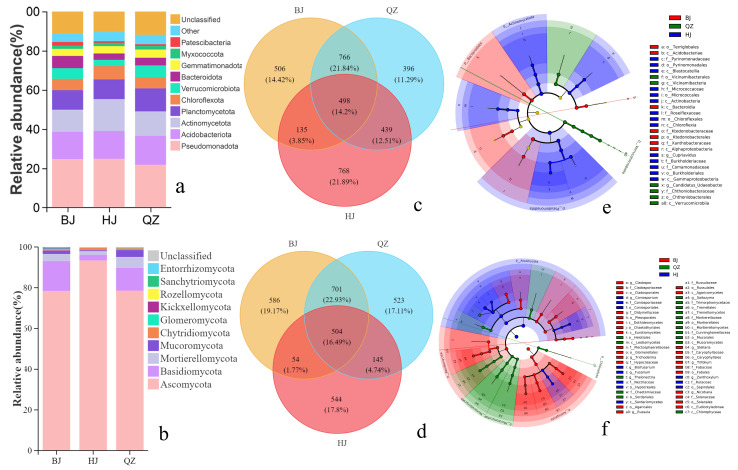
Soil microbial composition, OTU Venn diagrams, and LEfSe analysis in KSWCAF under different grades of KD. (**a**) Relative abundance of soil bacterial communities at the phylum level; (**b**) Relative abundance of soil fungal communities at the phylum level; (**c**) Bacterial OTU Venn diagram; (**d**) Fungal OTU Venn diagram; (**e**) Bacterial LEfSe analysis; (**f**) Fungal LEfSe analysis.

The relative abundances of the top 10 soil microbial phyla were analyzed (Figure 2a,b). The results showed that *Pseudomonadota*, *Acidobacteriota*, *Actinomycetota*, and *Planctomycetota* were the dominant bacterial phyla in the soil. The combined relative abundances of these dominant phyla at the BJ, QZ, and HJ sites were 59.9%, 60.78%, and 65.34%, respectively (Figure 2a, Table S1). Overall, the relative proportion of these dominant bacterial phyla tended to increase with increasing KD severity, although the differences were not statistically significant (Figure 2a, Table S1). Regarding the fungal community, the dominant phyla were *Ascomycota*, *Basidiomycota*, and *Mortierellomycota*, with *Ascomycota* being the most predominant. The *Ascomycota* OTUs accounted for 66.39%, 69.66%, and 85.19% of the total fungal OTUs at the BJ, QZ, and HJ sites, respectively (Figure 2b, Table S1). The relative abundance of *Ascomycota* increased with the severity of KD, whereas that of *Basidiomycota* decreased as desertification intensified (Figure 2b, Table S1). Venn diagrams were used to illustrate the similarities and differences among microbial OTUs from soil in the KD areas under integrated agroforestry management (Figure 2c,d). For the bacterial community, the numbers of unique OTUs in the BJ, QZ, and HJ sites were 506, 396, and 768, accounting for 14.42%, 11.29%, and 21.89% of the total OTUs, respectively. The number of OTUs shared among all three sites was 498, representing 14.2% of the total (Figure 2c). Specifically, the number of OTUs shared between BJ and QZ was 766 (21.84%), between QZ and HJ was 439 (12.51%), and between BJ and HJ was only 135 (3.85%). For the fungal community, the unique OTUs in BJ, QZ, and HJ were 586, 523, and 544, respectively, with 504 OTUs shared among all three sites (Figure 2d). The number of OTUs shared between BJ and QZ was 701, between QZ and HJ was 145, and between BJ and HJ was merely 54. This suggests that the degree of KD significantly influences the composition of the microbial community in this karst agroforestry ecosystem.

LEfSe was used to identify species with significant abundance differences within each group, namely, biomarkers (Figure 2e,f). At an LDA threshold of 3.8 or higher, 27 branches from kingdom to genus showed significant differences in soil bacteria, including 8 in BJ, 7 in QZ, and 16 in HJ (Figure 2e and Figure S2). Fifty-four branches showed significant differences for soil fungi, including 27 in BJ, 16 in QZ, and 11 in HJ (Figure 2f and Figure S2). Different bacterial biomarkers were more abundant in HJ (Figure 2e and Figure S2). This indicates that soil microbial structure and diversity in MHKD environments differ significantly from those in PL and LMKD environments. The significant enrichment of fungal biomarkers decreased with increasing KD grade, indicating that soil fungal structure and diversity are affected considerably by KD grade.

#### 3.2.2. Soil Microbial Diversity

**Figure 3:**
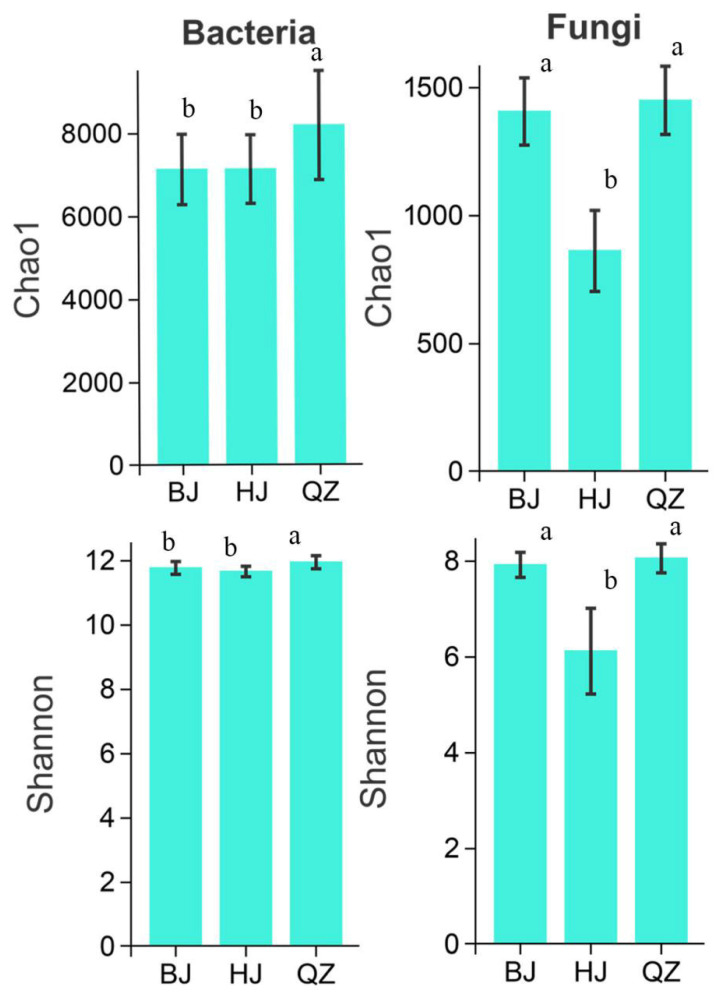
Chao and Shannon indices of soil microbial communities in KSWCAF across different KD grades. Different letters above the bars indicate significant differences in soil microbial α-diversity among KD grades (*p* < 0.05).

Chao and Shannon indices represent microbial community richness and diversity and were used to study α-diversity of soil bacteria and fungi in KSWCAF under different grades of KD (Figure 3). According to Figure 3, Chao indices for BJ, QZ, and HJ were 7136, 8192.07, and 7139.14, respectively, and Shannon indices were 11.78, 11.96, and 11.67, indicating that soil bacterial richness and evenness both showed a trend of first increasing then decreasing with worsening KD. Tukey’s HSD test revealed that both the Chao and Shannon indices for soil bacteria in QZ were significantly higher than in BJ and HJ (*p* < 0.05). At the same time, differences between BJ and HJ were not significant (Figure 3). This indicates that soil bacterial richness and evenness were highest in the LMKD environment, where the KSWCAF is healthier and more stable. For soil fungi, Chao indices for BJ, QZ, and HJ were 1407.07, 1450.05, and 862.71, respectively, and Shannon indices were 7.92, 8.06, and 6.12. Based on fungal α-diversity, the order of soil fungal richness and evenness under different grades of KD was LM > PL > MH. Tukey’s HSD test showed that both Chao and Shannon indices for soil fungi in HJ were significantly lower than in BJ and QZ (*p* < 0.05), while differences between BJ and QZ were not significant (Figure 3). This indicates that the MHKD environment is unfavorable for soil fungal survival.

**Figure 4:**
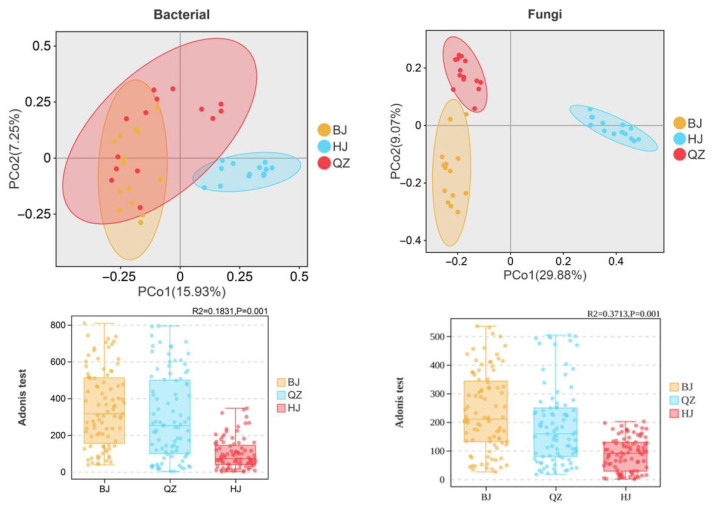
PCoA analysis and Permutational MANOVA test of soil microorganisms.

PCoA based on Bray–Curtis distance was used to analyze β-diversity of soil microorganisms in KSWCAF across different KD grades. Samples with similar community structures tended to cluster, while samples with significant community differences were far apart. PCoA results showed that OTUs in BJ and QZ overlapped considerably (Figure 4), whereas those in HJ were relatively independent. Permutational MANOVA test results based on Bray–Curtis distance also confirmed this finding (bacteria: R^2^ = 0.1831, *p* = 0.001; fungi: R^2^ = 0.3713, *p* = 0.001) (Figure 4). Community structure differences between groups were significant and larger than within-group differences, with fungal communities being more clustered than bacterial communities (Figure 4). This indicates that KD significantly affects soil microbial diversity in KSWCAF, with severe desertification having the most pronounced effect. Therefore, karst SWC efforts should focus on MHKD environments.

#### 3.2.3. Soil Microbial Co-Occurrence Network Characteristics Analysis

**Figure 5:**
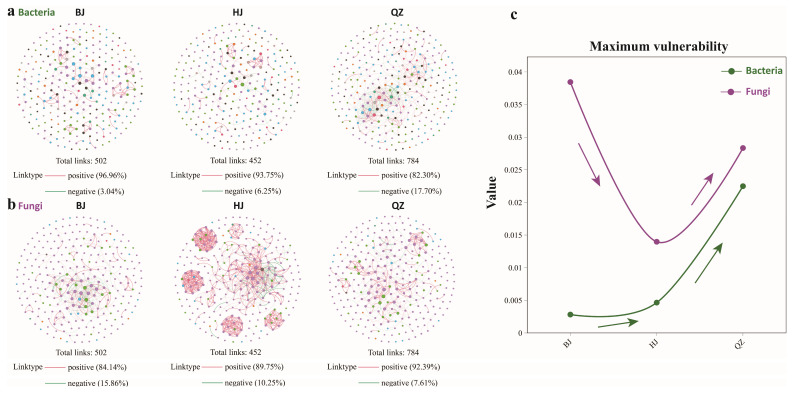
Microbial co-occurrence network diagrams ((**a**): Bacterial network characteristics; (**b**): Fungal network characteristics; (**c**): Microbial network vulnerability).

Significant differences were observed in the structural characteristics and stability of soil bacterial and fungal co-occurrence networks along the gradient of KD (Figure 5). In the bacterial network, the total number of edges was highest at the QZ stage (784), substantially exceeding those at the BJ (502) and HJ (452) stages (Table S2). This indicates that potential interactions among bacterial taxa were most frequent at this stage, with a marked increase in network complexity. However, this increase in complexity was accompanied by a significant rise in the proportion of negative correlations, which peaked at 17.70% during the QZ stage (Figure 5a). This suggests that under conditions of resource limitation and heightened environmental stress, competitive and antagonistic interactions among bacterial taxa intensified. This shift implies that at the LMKD, resource constraints and increased environmental heterogeneity may drive niche differentiation among bacterial taxa, thereby strengthening competitive relationships within the community. Furthermore, network vulnerability reached its maximum at the QZ stage. This indicates that although the bacterial network structure became more complex, its sensitivity to the loss of key nodes or external disturbances increased, leading to an overall decrease in stability. This pattern is characteristic of a “high complexity–low stability” state.

In contrast, the fungal co-occurrence network exhibited a different response pattern as KD intensified (Figure 5b). The total number of edges in the soil fungal network increased progressively with desertification severity, with 684, 749, and 1622 edges recorded for the BJ, QZ, and HJ stages, respectively (Table S2). This suggests a gradual increase in the complexity of the fungal network structure. Notably, at the HJ stage, which represents the most severe desertification (referred to as MHKD), the network displayed multiple highly aggregated modular substructures with tighter node connections (Figure 5b), indicating distinct characteristics of localized high-density connectivity. This observed increase in modularity suggests that under severe KD stress, the fungal community may enhance its resilience to environmental instability by forming relatively independent, internally highly cooperative functional units. Regarding interaction patterns, as the degree of KD increased, the overall proportion of positive correlations in the fungal network rose, while the proportion of negative correlations declined. The proportion of negative correlations was highest in the BJ stage (15.86%) and decreased to 7.61% and 10.25% in the QZ and HJ stages, respectively. Coupled with the formation of a distinct modular structure at the HJ stage (Figure 5c), these results indicate that synergistic relationships among fungal taxa were strengthened with increasing desertification. This likely reflects a strategy in which fungi cope with adverse environmental conditions by enhancing cooperation and functional complementarity. Further analysis revealed that the maximum vulnerability of the fungal network decreased along the desertification gradient, peaking at the BJ stage and being significantly lower at the HJ stage. This suggests that increased modularity and redundancy effectively enhanced network stability.

In summary, bacterial and fungal communities demonstrated markedly divergent responses along the KD gradient. The bacterial network reached its highest complexity at the LMKD stage, but this was accompanied by intensified competition and heightened vulnerability, indicating greater sensitivity to environmental change. In comparison, the fungal network enhanced its overall stability at the HJ stage through increased synergism and modular structure, reflecting a stronger capacity for environmental adaptation. This differential response suggests that in KD ecosystems, fungi may play a more critical role in maintaining the stability of the soil microbial network and the continuity of ecological functions.

#### 3.2.4. Functional Prediction Characteristics of Soil Microbes in SWCAF Under Different KD Grades

**Figure 6:**
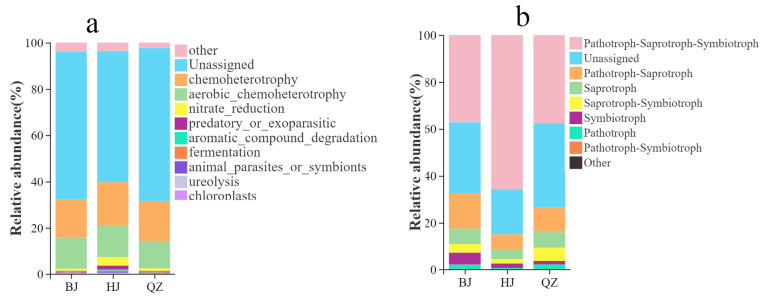
Prediction of fungal functions in soils under different grades of KD in KSWCAF ((**a**): Bacteria; (**b**): Fungi).

Using the FAPROTAX database, the functional profiles of soil bacterial communities in KSWCAF across different KD grades were predicted. The results revealed a distinct gradient response in the functional composition of the soil bacterial communities along the desertification gradient from BJ (PLKD) to QZ (LMKD) and then to HJ (MHKD) (Figure 6a). Across all three study areas, unassigned functions dominated the bacterial functional repertoire, with relative abundances exceeding 55%. This indicates a substantial presence of bacterial taxa with unknown functions in the KD ecosystem, reflecting the complexity and uncertainty of microbial functional composition in such environments. Among the assigned functions, chemoheterotrophy and aerobic chemoheterotrophy were the dominant functional types at all sites. Their relative abundances generally increased with increasing KD intensity, reaching their highest values at the HJ site. This suggests that under moderate to severe KD conditions, the soil bacterial community’s reliance on limited organic-carbon resources is significantly enhanced, with microbial metabolic activities increasingly focused on the decomposition and utilization of organic matter. This reflects an adaptive adjustment in soil carbon cycling in this degraded ecosystem. Furthermore, nitrogen-cycling-related functions showed a sensitive response to the KD gradient. Specifically, the nitrate reduction function was markedly enriched at the HJ site, whereas its proportion was lower at the BJ and QZ sites. This indicates that as the degree of KD intensifies, the pathways for soil nitrogen transformation tend to simplify, and the nitrogen-cycling process may be comprehensively influenced by factors such as soil structure fragmentation, altered moisture conditions, and micro-environmental hypoxia.

Using the FUNGuild database, functional predictions were generated for fungal communities in soils from KSWCAF under different degrees of KD. The results showed that fungi with mixed trophic modes were predominant across all KD grades. In the BJ, QZ, and HJ study areas, Pathotroph-Saprotroph-Symbiotroph was the functional type with the highest relative abundance, accounting for 37.18%, 37.61%, and 65.79% of the total community abundance, respectively, which was significantly higher than any single trophic mode group (Figure 6b). Unassigned functions also constituted a large proportion in all three study areas, with relative abundances of 30.22%, 35.81%, and 19.16%, respectively, indicating that a considerable portion of the fungal taxa could not be definitively classified at the trophic mode level. The relative abundances of single trophic mode groups, namely Saprotroph, Symbiotroph, and Pathotroph, were relatively low across all groups and showed only limited variation between different sites (Figure 6b). The HJ group exhibited the highest proportion of mixed trophic mode groups and the lowest proportion of single trophic mode groups. This suggests that community functions in this most degraded site may be concentrated among a few highly adaptive strategies, potentially at the cost of reduced functional diversity. In contrast, the BJ and QZ groups retained a proportion of single-trophic-mode groups, indicating a relatively more diverse functional structure, in which environmental filtering might be weaker or spatial heterogeneity might be higher (Figure 6b).

### 3.3. Drivers of Microbial Community Structure Changes in Karst Agroforestry Soils for SWC

**Figure 7:**
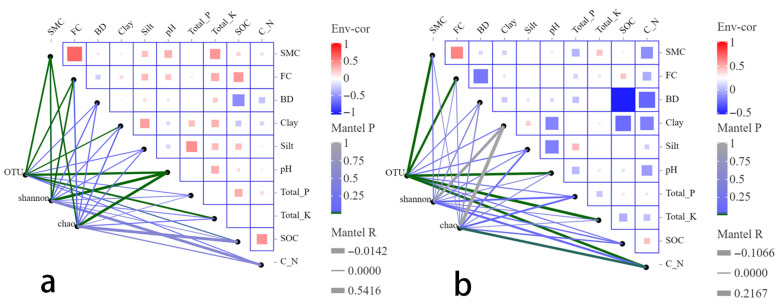
Mantel test of soil microbial OTUs and α-diversity. (**a**) Soil bacteria; (**b**) Soil fungi. The size of the blue and red boxes is proportional to the strength of the correlation.

Mantel test results revealed significant correlations between soil bacterial community OTU abundance, alpha-diversity indices (Shannon, Chao1), and various soil environmental factors, although the strength and direction of the responses varied considerably across indicators (Figure 7a). From the perspective of community composition, OTU-level community structure exhibited highly significant correlations with SMC, FC, pH, and Total_K (*p* < 0.01), and significant correlations with Clay and SOC (Table S3). The correlation coefficients (R^2^), ranked from highest to lowest, were pH (0.43), Total_K (0.30), SMC (0.27), FC (0.20), Clay (0.137), and SOC (0.111). At the alpha-diversity level, the Shannon and Chao1 indices showed some consistency in their responses to environmental factors, although the specific factors to which they responded differed. The Shannon index was highly significantly correlated with pH and significantly correlated with SMC and FC, whereas the Chao1 index was highly significantly correlated with pH and SMC, and significantly correlated with FC and Clay (Figure 7a, Table S3). This indicates that the evenness, complexity, and richness of the bacterial community were primarily regulated by soil physical properties.

Regarding the fungal community, at the OTU level, the OTU-level structure showed highly significant correlations with SMC, FC, pH, Total_K, and the C_N ratio (*p* < 0.01), with correlation coefficients of 0.195, 0.164, 0.191, 0.217, and 0.188, respectively. This suggests that the fungal community is regulated by multiple factors, including soil moisture status, pH, Total_Potassium availability, and the balance of carbon and nitrogen resources. In contrast, the correlation between soil texture and OTU composition was relatively weak (Figure 7b, Table S4). At the alpha-diversity level, aside from a significant correlation between the C_N ratio and the Chao1 index, no other environmental factors were significantly correlated with either the Shannon or Chao1 indices (*p* > 0.05). This indicates that the alpha diversity of soil fungi in the study area responded only weakly to direct influences from most environmental factors (Figure 7b, Table S5). This result suggests that, along the KD gradient, the fungal community maintains overall diversity stability through species replacement and functional redundancy, rather than responding to environmental changes via significant increases or decreases in species richness. Concurrently, the C_N ratio, as a key factor reflecting organic matter quality and resource stoichiometry, plays an important regulatory role in the potential species richness of the fungal community, implying that the carbon–nitrogen resource balance may be the primary ecological constraint limiting changes in fungal alpha diversity.

**Figure 8:**
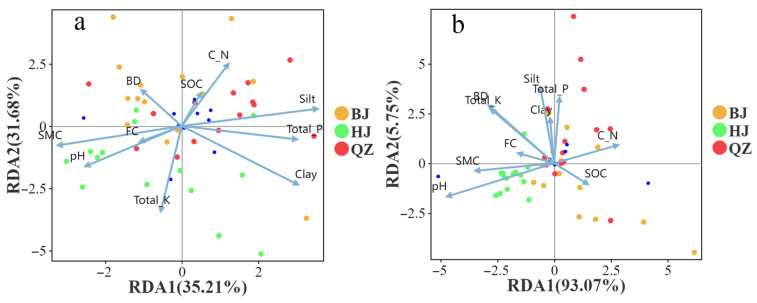
Redundancy analysis of the top 10 soil microbial phyla (blue dots) and soil properties (light blue arrows). (**a**): Bacteria; (**b**): Fungi. SMC: Soil moisture content; FC: Field capacity; BD: Bulk density; Total_P: Total_Phosphorus; Total_K: Total_Potassium; SOC: Soil organic carbon; C_N: Carbon–nitrogen ratio.

Redundancy analysis was conducted to examine the relationship between soil microbial community structure at the phylum level and soil physicochemical properties (Figure 8a,b). The results showed that soil physicochemical properties explained 66.89% of the total variation in soil bacterial community structure, with RDA1 and RDA2 accounting for 35.21% and 31.68%, respectively. Regarding site distribution, samples from the BJ area were mainly concentrated in the first and second quadrants, showing significant correlations with indicators such as BD, SOC, and C_N ratio. Samples from the QZ area were primarily located in the first and fourth quadrants, aligning with the direction of vectors for Silt, Total_P, and Clay. Samples from the HJ area were mainly clustered in the third and fourth quadrants, consistent with the direction of the pH, SMC, and FC vectors, indicating higher soil moisture content and pH in this area. Further analysis revealed that indicators such as Silt, Total_P, and Clay had high loadings on the RDA1 axis, serving as core variables driving sample differentiation along RDA1. Meanwhile, indicators such as pH, SMC, Total_K, and FC exhibited stronger explanatory power on the RDA2 axis, dominating the distribution pattern of samples along RDA2. This differentiation pattern suggests that distinct KD control areas shape distinct soil microenvironments by regulating key processes, such as soil texture, moisture status, and nutrient cycling, which may, in turn, exert profound influences on the structure and function of soil microbial communities.

Soil physicochemical properties collectively explained 98.82% of the total variation in soil fungal community structure, with RDA1 and RDA2 accounting for 93.07% and 5.75%, respectively. In terms of site distribution, samples from the BJ area were mainly concentrated in the fourth quadrant, showing significant correlations with indicators such as BD, SOC, and C_N ratio. Samples from the QZ area were primarily located in the first and fourth quadrants, aligning with the direction of vectors for Silt, Total_P, and Clay. Samples from the HJ area were mainly clustered in the third and fourth quadrants, consistent with the direction of the pH, SMC, and FC vectors, further confirming higher soil moisture content and pH in this area. Further analysis revealed that indicators such as Silt, Total_P, and Clay had high loadings on the RDA1 axis, serving as core variables driving sample differentiation along RDA1. Meanwhile, indicators such as pH, SMC, Total_K, and FC exhibited stronger explanatory power on the RDA2 axis, dominating the distribution pattern of samples along RDA2. This differentiation pattern suggests that distinct KD control areas shape distinct soil microenvironments by regulating key processes, such as soil texture, moisture status, and nutrient cycling, which, in turn, may exert profound influences on the structure and function of soil microbial communities.

**Figure 9:**
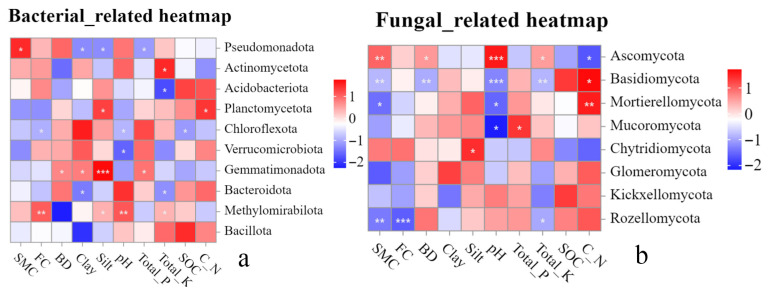
Heatmap showing the correlation between environmental factors and soil microbial communities at the phylum level (top 10). (**a**): Bacterial_related heatmap; (**b**): Fungi _related heatmap. Positive correlations are represented in red, and negative correlations in blue. The legend on the right displays the color scale corresponding to different correlation coefficients (*p*-values: * < 0.05; ** < 0.01; *** < 0.001). SMC: Soil moisture content; FC: Field capacity; BD: Bulk density; Total_P: Total_Phosphorus; Total_K: Total_Potassium; SOC: Soil organic carbon; C_N: Carbon–nitrogen ratio.

The related heatmap was used to reveal the relationship between environmental factors and soil microbial structure at the phylum level (top 10 in relative abundance) (Figure 9a,b). Overall, bacterial and fungal communities exhibited significantly differentiated responses to soil environmental factors (Figure 9). For soil bacteria, *Pseudomonadota* showed a significant positive correlation with SMC (*p* < 0.05) and significant negative correlations with Clay, Silt, and Total_P content. *Actinomycetota* exhibited a significant positive correlation with Total_K (*p* < 0.05), whereas *Acidobacteriota* showed a significant negative correlation with Total_K (*p* < 0.05), suggesting that potassium availability is a key factor driving the distribution of this phylum. *Planctomycetota* was significantly positively correlated with Silt and C_N ratio (*p* < 0.05), indicating its advantage in silt-textured soils and habitats with high carbon–nitrogen ratios. *Chloroflexota* displayed significant negative correlations with FC, pH, and SOC (*p* < 0.05). *Gemmatimonadota* showed an extremely significant positive correlation with Silt (*p* < 0.001) and significant positive correlations with BD, Clay, and Total_P (*p* < 0.05), highlighting the strong influence of soil texture, structure, and phosphorus content on its distribution. *Bacteroidota* was significantly negatively correlated with Clay and Total_K (*p* < 0.05). *Methylomirabilota* exhibited extremely significant positive correlations with FC and pH (*p* < 0.01) and significant positive correlations with Silt and Total_K (*p* < 0.05) (Figure 9a), suggesting that moisture conditions and pH are core environmental factors regulating its abundance.

Regarding soil fungi, *Ascomycota* showed extremely significant positive correlations with pH and SMC (*p* < 0.01), significant positive correlations with BD and Total_K (*p* < 0.05), but a significant negative correlation with C_N ratio (*p* < 0.05), indicating that this phylum prefers habitats with adequate moisture, compact structure, and high pH and Total_K levels. *Basidiomycota* exhibited a pattern opposite to that of *Ascomycota*, showing extremely significant negative correlations with SMC, BD, pH, and TotalK (*p* < 0.01), but a significant positive correlation with C_N ratio (*p* < 0.05). *Mortierellomycota* was significantly positively correlated with C_N ratio (*p* < 0.01) and significantly negatively correlated with SMC and pH (*p* < 0.05). *Mucoromycota* showed a significant positive correlation with Total_P (*p* < 0.05) and a significant negative correlation with pH (*p* < 0.05). *Chytridiomycota* was significantly positively correlated with Silt (*p* < 0.05). *Rozellomycota* exhibited extremely significant negative correlations with SMC and FC (*p* < 0.01) and a significant negative correlation with Total_K (*p* < 0.05).

## 4. Discussion

### 4.1. Effects of KD on Soil Properties in KSWCAF

It has long been believed that soil degradation increases with worsening KD, with the most severe soil degradation observed in high desertification environments [76,77]. However, this study shows significant differences in soil physicochemical properties among KD grades in SWCAF, with complex trends observed as KD severity increases (Table 2 and Table 3).

Results revealed significant differences in SMC, CWHC, FC, CP, clay, silt, sand, pH, Total_K, SOC, and C_N among different KD grades, indicating that these soil factors are significantly affected by KD. However, insignificant differences were observed in BD, NCP, TP, Total_P, Total_N, C_P, and N_P across different KD grades (*p* > 0.05). Meanwhile, CWHC and FC increased with worsening KD grade (Table 2), consistent with Sheng et al. [72], possibly because CP in PLKD environments was significantly lower than in LM and MHKD, while NCP was higher, resulting in substantially lower soil water-holding capacity in PLKD environments compared to LM and MHKD. BD showed an overall decreasing trend with increasing KD grade, with the highest in PLKD areas (Table 2), where soil-nutrient loss was more severe, consistent with previous studies [78], indicating the lowest soil-nutrient content in PLKD areas. LMKD has the lowest BD, though differences were not significant (*p* > 0.05) (Table 2), consistent with Lan et al. [79], Sheng et al. [72,78], but inconsistent with Long et al. [80]. This may relate to better socioeconomic conditions in the QZ study area, where farmers prioritize improving cropland soil fertility and engage in more intensive farming practices, such as intercropping and mixed cropping. In contrast, the BJ has lagging socioeconomic development, with traditional agricultural practices that have destroyed the original soil structure and increased erodibility. Clay and silt contents in BJ were significantly lower than in QZ and HJ, while differences between QZ and HJ were insignificant (Table 2). It may be related to reduced soil erosion with increasing KD grade [81]. LMKD environments had the highest proportions of silt and TP and the lowest BD, indicating that LMKD is conducive to improving soil physical properties in KSWCAF. PLKD environments had the lowest SMC, CWHC, FC, CP, and silt among all grades, and the highest BD, NCP, and sand, indicating the poorest soil physical quality.

Significant differences in soil chemical properties were observed among different grades of KD in SWCAF (Table 3). Soil pH is a crucial indicator of soil acidity and alkalinity, and it is vital for plant growth, nutrient absorption, microbial activity, and soil fertility. Our study showed that pH increased with worsening KD, being significantly higher in MHKD than in PL and LMKD, inconsistent with previous reports that moderate and severe desertification have lower pH than light desertification [77]. This may be because the exposed carbonate rock area increases with worsening KD, allowing greater contact area with atmospheric CO_2_ and precipitation, promoting carbonate rock dissolution and higher pH. Total_N is a reserve indicator of soil nitrogen nutrients, reflecting soil-nitrogen-supply capacity to some extent [82]. Total_P is the sum of all phosphorus forms in soil, including organic and inorganic phosphorus, and is an essential indicator of soil-phosphorus-supply capacity [83]. Both play crucial roles in agricultural production and ecosystems. This study found no significant differences in chemical indicators, such as Total_P and Total_N, across different KD grades (Table 3). This is consistent with previous studies [72], which indicate that karst soils are significantly influenced by carbonate rock parent material and are less affected by KD. Total_P is essential for plant photosynthesis, starch synthesis, and sugar conversion, and is an important indicator of soil fertility [84]. Total_K in PLKD was significantly (*p* < 0.05) lower than in LM and MHKD, consistent with previous studies [75]. Some studies have shown that SOC and Total_N contribute significantly to soil fertility in karst regions and can serve as sensitive indicators of the soil–KD relationship [85]. This study found that SOC and Total_N were higher in LMKD environments than in PL and MHKD environments, indicating that LMKD environments are conducive to improving soil fertility.

In summary, the quality of soil physicochemical properties in KSWCAF under KD ranked from high to low as LMKD > MHKD> PLKD environment. This conclusion is inconsistent with studies suggesting that soil physicochemical properties improve with increasing KD grade due to the aggregation effect of exposed rocks [72,79], and with studies suggesting that soil physicochemical properties worsen with increasing KD grade due to vegetation-cover effects [77]. This is because KD soils are influenced by multiple factors, including environmental factors (e.g., climate, parent material, topography) and anthropogenic factors (e.g., cultivation, grazing) [86]. The aggregation effect of exposed rocks may improve KD soil properties to some extent, but soil properties do not improve indefinitely with increasing KD grade; instead, there is a threshold. LMKD, with its specific vegetation cover and rock-exposure rate, supports litterfall that improves soil physicochemical properties. At the same time, the aggregation effect of exposed rocks also improves soil properties to some extent, resulting in optimal soil quality in LMKD environments.

### 4.2. Effects of KD on Soil Microbial Diversity in KSWCAF

A soil with a higher diversity index of bacteria and fungi is associated with greater resilience against environmental stressors, leading to more stable microecological function [87]. PCoA results showed substantial overlap in soil bacterial OTUs between BJ and QZ, while HJ was relatively independent (Figure 4). Permutational MANOVA results indicated a highly significant but small difference among soil bacteria in KSWCAF systems under different grades of KD (R^2^ = 0.1831, *p* = 0.001). PCoA results for soil fungi showed that samples from KSWCAF systems across different KD grades were independent of one another, each exhibiting a distinct pattern. Permutational MANOVA also revealed a highly significant moderate difference among them (R^2^ = 0.3713, *p* = 0.001). In summary, KD significantly affected soil microbial β-diversity. The Chao and Shannon indices, representing microbial community richness and diversity, were used to investigate the α-diversity of soil bacteria and fungi in KSWCAF systems under different KD grades (Figure 3). Our study found that both the Chao and Shannon indices of soil bacteria in the LMKD environment were significantly higher (*p* < 0.05) than those in the PLKD and MSKD environments. Furthermore, there were no significant differences (*p* > 0.05) in the bacterial Chao and Shannon indices between the latter two environments (Figure 3). This finding is inconsistent with that of Chen et al. [88], which suggested that the Shannon index and richness of the soil bacterial community decrease with increasing KD intensity. A possible explanation is that LMKD represents a transitional stage between PLKD and MSKD. During this transitional phase, ecosystems often exhibit a peak in biodiversity [89], thereby providing richer living resources for bacteria. Fungi, as decomposers, mutualists, or pathogens of soil animals and plants, influence community composition and productivity through material cycling and energy flow in ecosystems [11,90]. This study found that soil fungal diversity first increased and then decreased as KD intensified. The Chao and Shannon indices of soil fungi in the MS KD environment were significantly lower (*p* < 0.05) than those in PL and LMKD environments, while the difference between the latter two was not significant (*p* > 0.05) (Figure 3). This finding is consistent with previous studies reporting a gradual decline in soil fungal Shannon and richness indices with increasing KD intensity [91]. A possible reason is that the MSKD environment has a high bedrock exposure rate, with SOC significantly lower than that in LMKD (Table 3). Since fungi are microorganisms that primarily depend on SOC and suitable moisture, their growth and reproduction are strongly inhibited under such conditions. These results indicate that the MSKD environment severely restricts the survival of soil fungi, leading to community simplification where only extremely tolerant groups persist.

### 4.3. Effects of KD on Soil Microbial Co-Occurrence Networks in KSWCAF

Microorganisms interact within ecological niches through syntrophic, mutualistic, and competitive interactions [25,92], which are essential for the structure and dynamics of microbial communities [93,94,95]. Their co-occurrence patterns can be explored using association network analysis [96,97]. It has been widely recognized that the stability of soil ecosystems is closely associated with microbial community complexity, enhanced ecological functions, and an increased capacity to buffer environmental changes [98,99]. Generally, the stability of soil ecosystems is associated with increased complexity of soil microbial communities, enhanced soil microbial ecological functions, and improved buffering capacity against environmental changes. In this study, co-occurrence network analysis was employed to reveal potential interaction patterns among soil microbial taxa along the KD gradient and their responses to environmental stress. We found that bacterial and fungal networks exhibited significant differences in structural complexity, interaction types, and stability, reflecting fundamental differences in ecological strategies and environmental adaptability between the two microbial groups (Figure 5). In the bacterial network, the QZ stage had the highest number of edges, indicating that under LMKD conditions, potential connections among bacterial taxa were significantly enhanced. However, this increase in network complexity was accompanied by a simultaneous increase in the proportion of negative correlations and maximum vulnerability, exhibiting a typical “high complexity–low stability” characteristic. This phenomenon aligns with the complexity–stability theory proposed by May [100], suggesting that under resource-limited and increased environmental stress conditions, overly complex interaction networks are more susceptible to disturbance. Negative correlations are typically indicative of competition and antagonism. The increased proportion of negative correlations in bacterial networks with increasing KD grade reflects the process by which soil bacterial taxa reduce direct competition intensity through niche differentiation under resource-limited conditions [101]. Against the background of intensifying KD, soil-structure fragmentation, and unstable nutrient and water conditions, these factors may exacerbate competition among bacteria for carbon sources and inorganic nutrients, thereby leading to a decline in overall network stability. This suggests that bacterial communities in KSWCAF systems are highly sensitive to KD stress and that their network structure is more prone to functional dysregulation following the loss of key nodes.

In contrast, fungal co-occurrence networks exhibited distinctly different evolutionary pathways with increasing KD intensity. Although fungal alpha diversity generally decreased with environmental degradation, fungal co-occurrence networks displayed higher connectivity and stability, characterized by significant increases in edges and modularity, indicating a trend toward more centralized and robust network structures [102]. Modularity is one of the important mechanisms for enhancing ecological network stability, as it limits the spread of disturbances among modules, thereby increasing the overall system’s resistance to interference [103]. Furthermore, the proportion of positive correlations in fungal networks initially increased, then decreased with increasing KD grade. However, the proportion of positive correlations in MHKD was higher than that in PLKD, indicating that under stress conditions, fungal taxa tend to maintain community stability through synergistic interactions and functional complementarity. This characteristic is closely related to the hyphal structure of fungi and their ability to transfer resources across scales, conferring greater environmental adaptability in degraded ecosystems [13]. Overall, although fungal species richness declined in environmentally degraded areas, fungal co-occurrence networks exhibited higher connectivity and stability, suggesting a trend toward a more centralized and robust network structure [102]. This may be because all dominant plants in the three study areas can form arbuscular mycorrhizae, and arbuscular mycorrhizal fungi are generally host-nonspecific, potentially resulting in lower diversity. Meanwhile, the scarcity of ectomycorrhizae, coupled with the dominance of Ascomycota, suggests that fungi have a significant influence on saprotrophic functions, alongside some opportunistic parasitism, while having a lesser impact on symbiotic functions. This may also be one of the reasons for the fungi’s higher stability. Additionally, the dominance of mixed trophic groups may promote stronger and more numerous interactions, thereby enhancing network connectivity and structural stability, even as species diversity declines [104,105]. These results indicate that environmental stress, by filtering for taxa with high tolerance and interaction capacity, drives the reorganization of fungal communities toward functional convergence and network integration, thereby maintaining the ecosystem’s functional potential.

### 4.4. Prediction of Soil Microbial Functions

Bacterial functional predictions based on FAPROTAX revealed a high proportion of unassigned functions in soils across all grades of KD, reflecting the high complexity and uncertainty of microbial functional composition in KD ecosystems. Among the assigned functions, chemoheterotrophy and aerobic chemoheterotrophy dominated across all sites and showed an increasing trend with increasing KD severity, indicating that under MHKD conditions, bacterial metabolic activities are increasingly focused on the decomposition and utilization of limited organic carbon resources. This trend aligns with the general principle that “carbon limitation driving enhanced heterotrophic metabolism” in degraded ecosystems [106]. Nitrogen-cycling-related functions, particularly the significant enrichment of nitrate reduction at the HJ stage, suggest that soil nitrogen transformation pathways tend to simplify as KD intensifies. This phenomenon may be associated with changes in soil pore structure, heterogeneous moisture distribution, and the formation of localized hypoxic microenvironments, which promote denitrification-related processes [107]. This result indicates that KD not only affects microbial community structure but also profoundly impacts soil nutrient cycling processes by altering functional composition.

FUNGuild analysis revealed that mixed trophic modes (Pathotroph–Saprotroph–Symbiotroph) dominated across all grades of KD and increased with increasing desertification severity, reaching a relative abundance of 65.79% at the MHKD stage (Figure 6b). This indicates that the functional structure of fungal communities tends to be dominated by taxa with broad ecological niches, exhibiting a clear pattern of functional convergence [105]. This phenomenon likely occurs because mixed trophic mode fungi possess high ecological flexibility, enabling them to switch between survival strategies under different resource conditions and host dependencies [108]. Therefore, their enrichment at the MHKD stage reflects a shift in functional diversity toward “convergence on highly adaptive strategies” amid intensified environmental filtering pressure. Meanwhile, the relative abundance of single trophic mode groups remained consistently low across all groups, consistent with the view that environmental filtering weakens functionally specialized taxa [104].

### 4.5. Effects of Environmental Factors on Soil Microorganisms

The structure and composition of microbial communities changed as the habitat changed [109,110]. Different microbial groups exhibit differential responses to the same environmental factors [111]. KD affects soil physicochemical properties [78], thereby altering microbial diversity and composition. RDA results showed that soil physicochemical properties explained 66.89% and 98.82% of the variation in bacterial and fungal communities, respectively (Figure 8), indicating that changes in community composition were highly correlated with the soil environmental gradient. This aligns with the conclusion by Chi et al. [57] that “microbial community structure is primarily controlled by soil environmental factors.”.

Our study found that bacteria responded more “rapidly” and “sensitively” to soil physicochemical properties. Mantel test revealed that bacterial community composition (OTU level) was highly significantly correlated with pH, Total_K, SMC, and FC, with pH showing the highest explanatory power (R^2^ = 0.43), suggesting that pH is the primary environmental filter driving bacterial community reassembly. Meanwhile, bacterial Shannon and Chao1 indices were also significantly or highly significantly correlated with pH, SMC, FC, and Clay (Figure 7a, Table S3), indicating that bacterial diversity (evenness and potential richness) is more sensitive to moisture, pH, and soil texture. This is consistent with numerous studies showing that bacteria have relatively narrow optimal pH ranges and that community composition undergoes rapid turnover along pH gradients [112]. From an ecological strategy perspective, bacterial communities often contain more taxa that respond quickly to resources; when water availability, ionic strength, and mineral nutrients (e.g., K) change, their metabolism and reproduction can be rapidly “amplified,” resulting in significant fluctuations in community structure and α-diversity [113].

Unlike bacteria, fungal α-diversity showed weaker direct responses to most environmental factors. Except for a significant correlation between C_N and the Chao index, other factors were mostly not significantly correlated with the Shannon and Chao indices. However, at the OTU composition level, fungal communities were highly significantly correlated with SMC, FC, pH, Total_K, and C_N (Figure 7b, Table S3), indicating that fungi tend to maintain overall diversity stability through “species replacement/functional compensation” rather than substantial fluctuations in diversity indices in response to environmental changes. This pattern aligns with the view that “fungal hyphal networks and functional redundancy enhance system stability,” which suggests that fungi can forage and transport resources across microhabitats via mycelium, thereby reducing sensitivity to local short-term fluctuations [114].

Regarding dominant phyla, different bacterial and fungal taxa exhibited differential sensitivities to soil properties [21]. *Pseudomonadota* showed a significant positive correlation with SMC (*p* < 0.05) and significant negative correlations with Clay, Silt, and Total_P content. This may be because *Pseudomonadota* are aerobic bacteria, and the high porosity and strong aeration of sand enhance oxygen availability, thereby promoting their rapid proliferation [113]. In contrast, clay and silt have lower porosity, limiting bacterial movement and oxygen diffusion and thereby inhibiting aerobic bacterial growth. Additionally, *Pseudomonadota* are predominantly r-strategists, preferring low-nutrient, high-disturbance environments. In this study, soil-nutrient levels across the three study areas followed the order QZ > HJ > BJ (Table 3). The relative abundance of *Pseudomonadota* was lowest in the QZ area, while *Acidobacteriota* was highest in the QZ area (Table S1). The significant correlations between *Acidobacteriota* and Total_N, SOC, C_P, and N_P (Figure 9b) further demonstrate that high nutrient content favors *Acidobacteriota* survival while limiting *Pseudomonadota* proliferation. This also suggests that in low-nutrient areas affected by KD, inoculation with *Pseudomonadota* could promote vegetation colonization and facilitate ecological restoration.

We also observed that the dominant fungal phylum *Ascomycota* was highly significantly positively correlated with pH and SMC, significantly positively correlated with BD and Total_K, and significantly negatively correlated with C_N (Figure 9b). This may be attributed to the fact that surface soils in karst slopes, due to developed rock fissures, have significantly higher capillary porosity than non-karst areas. Capillary porosity not only retains water [115] but also improves soil aeration, reducing anaerobic microenvironments. *Ascomycota*, being aerobic fungi, require high moisture environments for hyphal growth and spore germination, thus showing significant positive correlations with SMC, CWHC, and other soil physical properties. Furthermore, the significant positive correlation between *Ascomycota* and Total_K is consistent with previous studies [115,116], potentially because potassium ions, as cofactors for various enzymes (e.g., fungal amylase, protease), directly enhance the catabolic efficiency of *Ascomycota*. However, the significant negative correlation between *Ascomycota* and the C_N ratio (Figure 9b) aligns with findings from permafrost studies in the Bell River region [117]. This may be because *Ascomycota* preferentially decompose easily degradable organic matter (e.g., low C_N litter), while soils with high C_N ratios contain higher proportions of recalcitrant carbon (e.g., lignin), which is decomposed less efficiently [115,116]. In contrast, *Basidiomycota* showed highly significant negative correlations with SMC, BD, pH, and Total_K, but a significant positive correlation with C_N (Figure 9b). This is consistent with previous studies [118,119], possibly because *Basidiomycota* prefer moderately dry, well-aerated environments, and fungi are generally most active under weakly acidic to neutral conditions [118]. Additionally, geogenic potassium (total_K) released from karst parent rock weathering is high but poorly available.

In summary, our study revealed that the relative abundance of the dominant bacterial phylum *Pseudomonadota* was lowest in the QZ area, whereas *Acidobacteriota* was highest there. The dominant fungal phylum *Ascomycota* increased with intensifying KD (Figure 2). This may be because *Pseudomonadota*, as r-strategists, prefer pulsed nutrient supply; nutrient homeostasis in the QZ area suppressed their abundance. *Acidobacteriota*, as K-strategists, are better adapted to stable nutrient environments, and the high SOC, C_N, and clay content in QZ provided favorable conditions, resulting in their highest relative abundance in this area. Regarding fungi, the increasing relative abundance of *Ascomycota* with intensifying KD may be attributed to their better adaptability to moisture fluctuations and higher pH (Table 3). *Ascomycota* possess strong drought tolerance and broad substrate adaptability, enabling them to rapidly occupy niches under water-limited and relatively high pH conditions [120]. Furthermore, as KD intensifies, environmental stress filters for more tolerant taxa, promoting *Ascomycota* as the dominant group.

### 4.6. Limitations and Prospects

This study employed a space-for-time substitution approach, using spatial sequences of different KD grades to approximate the temporal process of KD evolution and explore changes in soil microbial community structure and functions along an intensifying KD gradient. Therefore, the causal inferences regarding thresholds in soil microbial and physicochemical properties presented in this study are tentative. Future research should incorporate long-term temporal studies to accurately characterize the patterns of soil microbial changes associated with KD grade evolution. Additionally, since KD grades are spatially “coupled” to specific study areas (i.e., each grade is represented by only one region), it is impossible to completely distinguish between “KD effects” and “regional effects” (i.e., inherent geographical and climatic differences among regions). Consequently, the conclusions focus on describing observed correlations and trends rather than definitive causal effects. Subsequent studies should further validate the universality of these findings by establishing replicated sampling areas across multiple independent regions. Furthermore, this study lacks an analysis of Archaeal community composition, abundance, and their correlations with environmental factors. Given that Archaea are particularly important in karst environments, future research should specifically address their potential roles in karst soil ecosystems. Finally, some Mucoromycota may well be the “fine endophytes”—primitive arbuscular-type mycorrhizae that are often found in more extreme environments. This is well worth following up with in future research.

## 5. Conclusions

(1) KD significantly altered the soil physicochemical properties of KSWCAF systems, but the changes exhibited a phased rather than linear decreasing trend. At the LMKD stage, the combined effects of rock aggregation and vegetation cover resulted in higher soil moisture, SOC, and C_N ratios, leading to optimal overall soil quality.
(2) KD significantly reshaped soil microbial community structure and β-diversity. Bacterial α-diversity peaked at the LMKD stage, whereas MHKD significantly reduced fungal richness and evenness, indicating stronger filtering effects on fungal taxa in severely degraded environments.
(3) Co-occurrence network analysis revealed that bacterial networks exhibited the highest complexity but increased vulnerability at the LMKD stage, characterized by “high complexity–low stability.” In contrast, fungal networks enhanced overall stability at the MHKD stage by increasing modularization and synergistic interactions, thereby demonstrating greater environmental adaptability.
(4) Soil physicochemical properties were the core factors driving microbial community assembly, with pH, moisture conditions, and C_N stoichiometry serving as key regulatory variables. Bacteria responded more sensitively to environmental gradients, while fungi maintained system stability through species substitution and functional redundancy.

In summary, KSWCAF ecosystems under KD conditions exhibited significant microbial reorganization and functional adjustment processes. It is recommended to use the optimal texture and nutrient window in LMKD areas (QZ) to develop high-efficiency, economically viable forests and fruit trees. For MHKD areas (HJ), the alkalization threshold of pH > 7.0 and the low fertility baseline of SOC < 24 g/kg indicate that the system has entered an “intervention warning zone” for ecological restoration. Priority should be given to deploying calcium-loving and alkali-tolerant species (e.g., *Zanthoxylum bungeanum*, *Broussonetia papyrifera*) and to implementing water-saving measures. Additionally, key microbial taxa should be integrated into KD monitoring systems, promoting a social-ecological synergy model of “ecological restoration + industrial cultivation + community participation” to provide a scientific basis and practical pathways for sustainable development in karst regions. This study provides microbiological theoretical support for implementing SWC and ecological restoration tailored to local conditions in karst areas.

## Figures and Tables

**Table 1:** Classification standards for karst desertification grades [68].

Karst Desertification Grade	Bedrock Exposure Rate in 0.2 km^2^ Patch (%)	Vegetation + Soil Cover Rate in 0.2 km^2^ Patch (%)	Reference Indicators
No desertification	<20	>80	Non-terraced sloping farmland with slope ≤ 15°, field dams, construction land, etc., good ecological environment; dense forest-shrub-grass vegetation; no obvious soil erosion; suitable for agriculture, forestry, and animal husbandry.
Potential desertification	20~30	80~70	Non-terraced dry sloping land with slope > 15°, grassland, etc., sparse forest-shrub-grass vegetation; good soil formation conditions but obvious soil erosion; trend of bedrock exposure.
Light desertification	31~50	69~50	Initial bedrock exposure; obvious soil erosion; low community structure dominated by sparse shrubs and grasses, or artificial dryland vegetation.
Moderate desertification	51~70	49~30	Intensified rocky desertification; severe soil erosion; shallow soil layer; mostly rocky sloping farmland and sparse shrub-grass slopes.
high desertification	71~90	29~10	Strong rocky desertification; basically no soil loss; mostly unusable land on the verge of losing agricultural value.
Extreme desertification	>90	<10	Complete rocky desertification; no soil loss on the surface; complete loss of agricultural value; typical unusable land.

**Table 2:** Soil physical properties in SWCAF under different grades of KD.

Site	SMC	CWHC	FC	BD	NCP	CP	TP	Clay	Silt	Sand
	%	%	%	g/cm^3^	%	%	%	%	%	%
BJ	10.75 ± 3.12 b	16.85 ± 2.90 b	15.85 ± 2.55 b	1.11 ± 0.18 a	38.71 ± 9.34 a	18.58 ± 4.08 b	57.28 ± 5.93 a	9.05 ± 3.83 b	57.28 ± 8.69 b	33.67 ± 9.76 a
QZ	11.78 ± 1.52 b	18.89 ± 1.44 a	18.01 ± 1.16 a	1.09 ± 0.13 a	37.21 ± 6.47 a	20.63 ± 2.42 ab	57.83 ± 4.38 a	11.60 ± 2.66 a	64.71 ± 3.42 a	23.69 ± 5.21 b
HJ	14.46 ± 2.22 a	19.73 ± 2.98 a	18.23 ± 3.09 a	1.10 ± 0.14 a	35.90 ± 6.83 a	21.61 ± 3.21 a	57.51 ± 4.71 a	10.56 ± 1.97 ab	58.82 ± 9.47 ab	30.62 ± 11.07 a

Note: SMC: soil water content; CWHC: capillary water holding capacity; FC: field capacity; BD: bulk density; NCP: non-capillary porosity; CP: capillary porosity; TP: Total_Porosity; Values within the same column not sharing the same lowercase letter indicate significant differences between samples (*p* < 0.05).

**Table 3:** Soil C, N, and P concentrations and stoichiometric characteristics in SWCAF under different grades of KD.

	pH	Total_P	Total_K	Total_N	SOC	C_N	C_P	N_P
		g/kg	g/kg	g/kg	g/kg	g/kg	g/kg	g/kg
BJ	6.46 ± 0.77 b	0.75 ± 0.29 a	6.06 ± 1.60 b	2.46 ± 0.71 a	26.45 ± 10.61 ab	10.51 ± 1.49 ab	41.58 ± 30.16 a	3.74 ± 2.01 a
QZ	6.70 ± 0.63 b	0.92 ± 0.19 a	14.32 ± 4.80 a	2.77 ± 0.62 a	33.85 ± 11.82 a	11.99 ± 1.50 a	38.11 ± 15.66 a	3.13 ± 0.97 a
HJ	7.22 ± 0.31 a	0.74 ± 0.27 a	14.56 ± 2.95 a	2.43 ± 0.67 a	23.71 ± 9.52 b	9.65 ± 2.79 b	33.69 ± 11.93 a	3.58 ± 1.21 a

Note: All values are reported as “mean ± standard deviation” based on measurements from 14 samples per study area. Values within the same column not sharing the same lowercase letter indicate significant differences between samples (*p* < 0.05). Total_P: Total_Phosphorus; Total_K: Total_Potassium; Total_N: total nitrogen; SOC: soil organic carbon; C_N: carbon nitrogen ratio; C_P: carbon phosphorus ratio; N_P: nitrogen phosphorus ratio.

## Data Availability

The datasets generated during the current study are not publicly available as they were collected by the authors, but they are available from the corresponding author on reasonable request.

## Supplementary Materials

The following supporting information can be downloaded at: https://www.mdpi.com/article/10.3390/microorganisms14030556/s1, Figure S1. Screening of environmental factors. Figure S2. Histogram of the linear discriminant analysis (LDA) scores (*p* < 0.05; LDA score 3.8). Table S1. Statistical table of the relative abundance of the top 10 microbial phyla in KSWCAF under different degrees of KD. Table S2. Microbial network characteristics of KSWCAF across different KD levels. Table S3. Hierarchical strategy for topological structure rationality validation. Table S4. Mantel test analysis of correlations between environmental factors and soil bacterial communities. Table S5 Mantel test analysis of correlations between environmental factors and soil fungal communities. Table S6. List of abbreviations.
