# Supplementary material for: Response of Soil Microbial Communities to Karst Desertification in Soil and Water Conservation Agroforestry Systems
Source: Microorganisms. 2026 Feb 28;14(3):556. doi: 10.3390/microorganisms14030556 (PMC13028859; doi:10.3390/microorganisms14030556)
Supplement: Supplementary file 1 [file microorganisms-14-00556-s001.zip › microorganisms-4149779-supplementary.pdf]

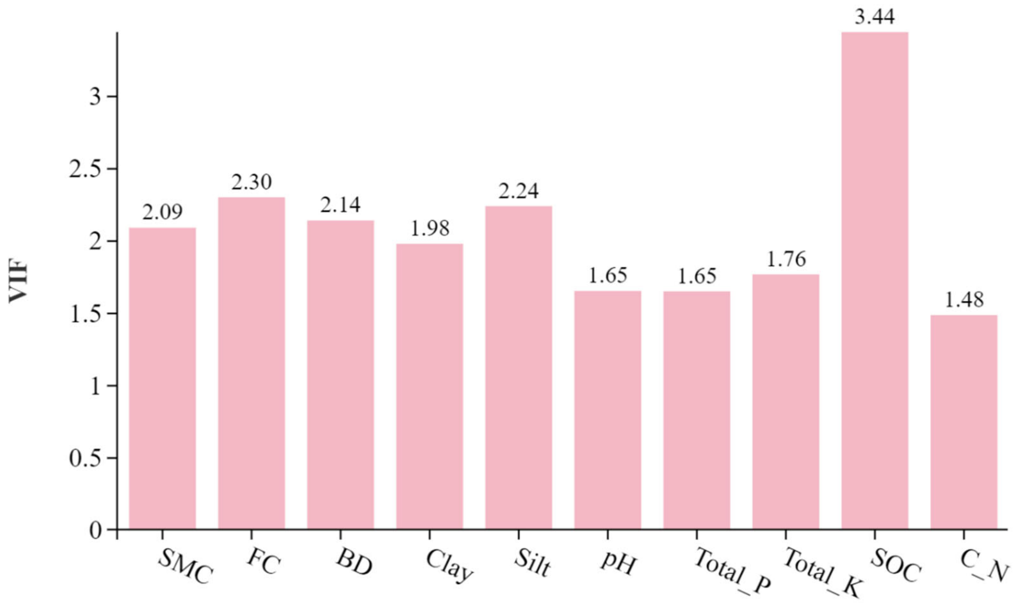

Figure S1. Screening of environmental factors.

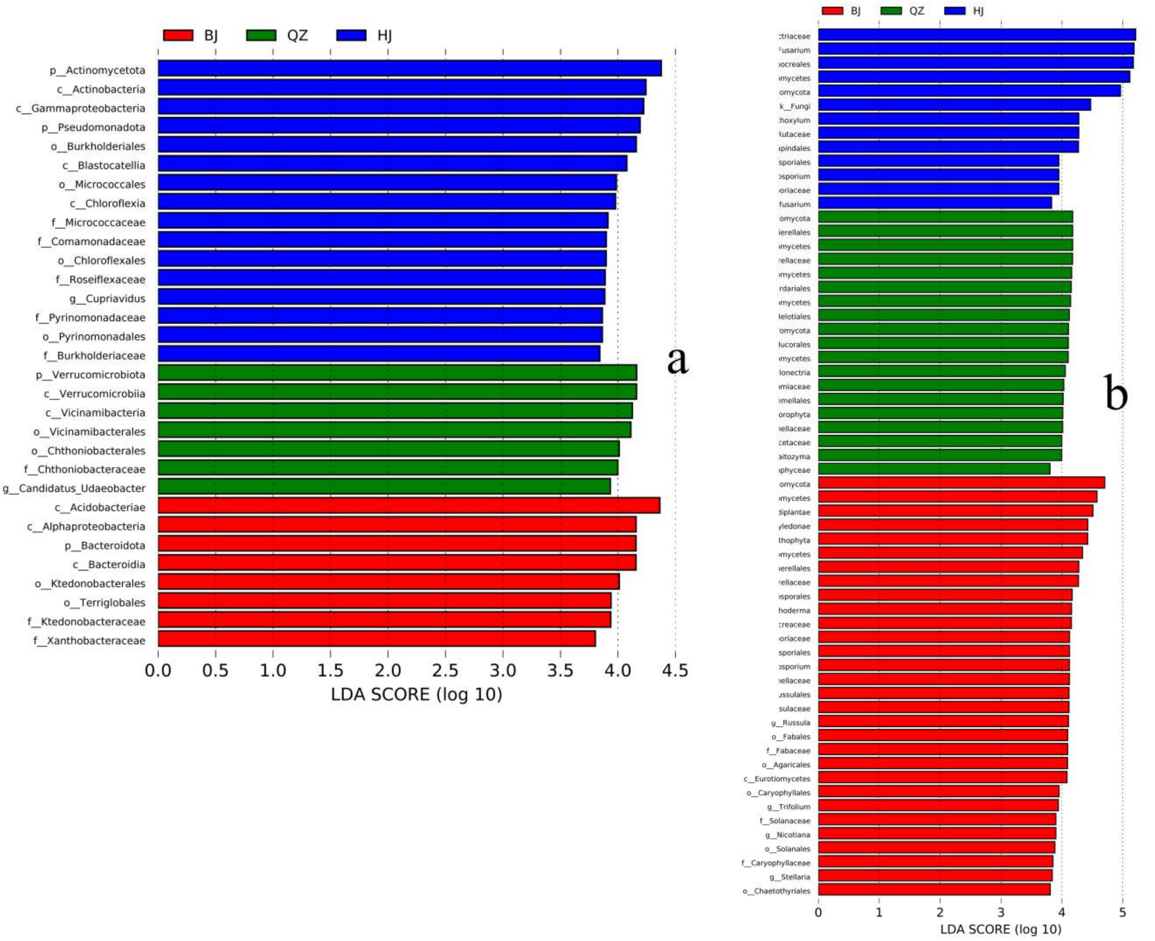

Figure S2. Histogram of the linear discriminant analysis (LDA) scores (P < 0.05; LDA score 3.8)

Table S1. Statistical table of the relative abundance of the top 10 microbial phyla in KSWCAF under different degrees of KD

Bacteria（%）				Fungi（%）			
Species taxonomy	BJ	QZ	HJ	species taxonomy	BJ	QZ	HJ
Pseudomonadota	24.74	21.79	24.89	*Ascomycota*	66.39	69.66	85.19
Acidobacteriota	13.92	14.78	14.21	*Basidiomycota*	13.09	10.50	2.76
Actinomycetota	11.26	12.45	16.24	*Mortierellomycota*	10.24	5.46	5.81
Planctomycetota	9.98	11.76	10.06	*Mortierellomycota*	2.92	4.62	1.67
Chloroflexota	5.73	5.75	6.78	*Chlorophyta*	2.65	2.80	0.73
Verrucomicrobiota	5.48	5.98	3.18	*Mucoromycota*	1.46	2.97	0.50
Bacteroidota	6.21	3.92	3.22	*Chytridiomycota*	0.69	0.89	1.14
Gemmatimonadota	3.61	4.18	3.78	*Ciliophora*	0.52	1.24	0.96
Myxococcota	1.59	1.83	1.68	*Glomeromycota*	0.54	0.52	0.26
Patescibacteria	1.94	0.83	0.81	*Kickxellomycota*	0.37	0.05	0.04

Table S2. Microbial network characteristics of KSWCAF across different KD levels

microbe	Bacteria			Fungi		
	BJ	QZ	HJ	BJ	QZ	HJ
KD 等级	PLKD	LMKD	MHKD	PLKD	LMKD	MHKD
Nodes	273	286	269	268	277	269
Edges	502	784	452	684	749	1622
Average degree	3.136	2.974	5.098	4.612	7.978	4.65
Average path length	4.35	2.604	4.703	2.997	3.63	5.687
Network diameter	13	7	15	8	10	12
Network density	0.012	0.011	0.0018	0.017	0.03	0.017
Clustering coefficient	0.781	0.825	0.718	0.772	0.789	0.724
Modularity	0.909	0.884	0.653	0.654	0.759	0.812

Table S3 Hierarchical strategy for topological structure rationality validation

Analysis Stage	FDR Threshold	Purpose	Rationale
Network construction	0.01	Control false positive edges (spurious correlations)	Ensure high reliability of network backbone
Vulnerability calculation	0.05	Retain potentially key nodes	Avoid omission of biologically important information

Table S4 Mantel test analysis of correlations between environmental factors and soil bacterial communities

Soil physical and chemical properties	OTU			Shannon			Chao		
	r^2	p	Sig.	r^2	p	Sig.	r^2	p	Sig.
SMC	0.272	＜0.01	**	0.222	0.016	*	0.211	⩽0.01	**
FC	0.195	＜0.01	**	0.250	0.013	*	0.180	0.046	*
BD	0.078	0.066		0.143	0.066		0.130	0.075	
Clay	0.137	0.012	*	0.098	0.142		0.177	0.048	*
Silt	0.079	0.108		0.095	0.177		0.109	0.152	
pH	0.428	＜0.01	**	0.542	＜0.01	**	0.519	＜0.01	**
Total_P	0.052	0.131		0.037	0.28		0.001	0.43	
Total_K	0.30	＜0.01	**	0.054	0.222		0.095	0.128	
SOC	0.111	0.036	*	0.019	0.358		-0.016	0.522	
C_N	0.079	0.07		-0.014	0.512		-0.004	0.482	

Table S5 Mantel test analysis of correlations between environmental factors and soil fungal communities

Soil physical and chemical properties	OTU			Shannon			Chao		
	r^2	p	Sig.	r^2	p	Sig.	r^2	p	Sig.
SMC	0.195	＜0.01	**	0.045	0.276		-0.004	0.508	
FC	0.164	＜0.01	**	0.004	0.425		0.016	0.398	
BD	0.028	0.218		-0.030	0.565		-0.031	0.61	
Clay	0.04	0.193		-0.107	0.904		-0.142	0.986	
Silt	0.052	0.151		0.033	0.314		0.107	0.129	
pH	0.191	＜0.01	**	-0.011	0.46		0.018	0.354	
Total_P	0.039	0.217		0.120	0.08		0.118	0.069	
Total_K	0.217	＜0.01	**	0.017	0.358		0.006	0.386	
SOC	0.041	0.185		0.076	0.196		0.077	0.185	
C_N	0.188	＜0.01	**	0.153	0.065		0.193	0.025	*

Table S6. List of abbreviations

Serial Number	Original Name	Abbreviation
1	krast desertification	KD
2	soil and water conservation agroforestry systems	SWCAF
3	karst soil and water conservation agroforestry systems	KSWCAF
4	potential-light	PL

5	light-moderate	LM
6	moderate–high	MH
7	moderate–high krast desertification	MHKD
8	potential-light krast desertification	PLKD
9	light-moderate krast desertification	LMKD
10	Soil and water conservation	SWC
11	average path length	APL
12	network diameter	ND
13	agroforestry systems	AF
